# A diagnostic approach to neurocutaneous syndromes

**DOI:** 10.1055/s-0045-1809664

**Published:** 2025-06-25

**Authors:** Sofia Mônaco Gama, João Vitor Gerdulli Tamanini, Marianna Pinheiro Moraes de Moraes, Thiago Yoshinaga Tonholo Silva, Fernanda Teresa de Lima, José Luiz Pedroso, Orlando Graziani Povoas Barsottini

**Affiliations:** 1 Universidade Federal de São Paulo, Escola Paulista de Medicina, Departamento de Neurologia e Neurocirurgia, São Paulo SP, Brazil.; 2 Universidade Federal de São Paulo, Escola Paulista de Medicina, Departamento de Ginecologia, São Paulo SP, Brazil.

**Keywords:** Neurocutaneous Syndromes, Neurofibromatoses, Tuberous Sclerosis

## Abstract

Neurocutaneous syndromes are a group of genetically and phenotypically diverse disorders that primarily affect the skin, central and peripheral nervous systems, and eyes. Classifying neurocutaneous syndromes based on genetic mechanisms often proves impractical in routine clinical settings. This review proposes a practical classification of neurocutaneous syndromes based on their neurological manifestations, including neoplastic lesions, epilepsy, vascular abnormalities, and ataxia. In this narrative review, we examined original articles and reviews that explore neurocutaneous syndromes, published between January 2000 and July 2024. The figures are part of a personal collection of the authors. Early recognition of dermatological and neurological hallmarks can guide diagnosis and prompt timely evaluation and treatment. Therefore, a thorough understanding of neurocutaneous syndromes highlights the importance of integrated diagnostic strategies that combine neurological and dermatological assessments.

## INTRODUCTION

Neurocutaneous syndromes, historically referred to as phacomatoses, represent a diverse group of genetically distinct multisystem disorders primarily affecting the skin, central and peripheral nervous systems, and eyes. 1 These syndromes exhibit considerable phenotypic and genetic variability, a reflection of the shared embryonic ectodermal origin of the nervous system and skin. 1 2

Advances in genetic research have significantly deepened our understanding of the pathophysiology underlying these disorders. However, classifying neurocutaneous syndromes solely based on genetic mechanisms often proves impractical in routine clinical settings. To address this limitation, this review proposes a pragmatic classification system centered on predominant neurological manifestations.

This review will explore major neurocutaneous syndromes frequently encountered in clinical practice, including neurofibromatosis types I and II (NF1 and NF2), von Hippel-Lindau (VHL) syndrome, tuberous sclerosis complex (TSC), hypomelanosis of Ito (HI), Sturge-Weber Syndrome (SWS), hereditary hemorrhagic telangiectasia (HHT), ataxia-telangiectasia, and cerebrotendinous xanthomatosis. Some less common conditions, such as Gorlin-Goltz Syndrome (GGS) and lipoid proteinosis (LP), will also be discussed. Given the broad spectrum of these disorders, neurologists must incorporate comprehensive dermatological evaluations into routine clinical assessments, as these can provide critical diagnostic insights.

Neurocutaneous syndromes are grouped into four main clinical categories: oncological manifestations, epilepsy, vascular abnormalities and ataxia. This framework aims to facilitate a structured and practical approach to the diagnosis and management of these complex conditions.

In this narrative review, we scrutinized original articles and reviews that explore neurocutaneous syndromes. To identify relevant studies, we used a combination of keywords and medical subject headings (MeSH) terms specifically designed to encompass the major neurocutaneous syndromes, including *neurocutaneous syndromes, phacomatoses, neurofibromatosis type I, neurofibromatosis type II, von Hippel-Lindau syndrome, tuberous sclerosis complex, hypomelanosis of Ito, Sturge-Weber syndrome, hereditary hemorrhagic telangiectasia, ataxia-telangiectasia, cerebrotendinous xanthomatosis, Gorlin-Goltz syndrome and lipoid proteinosis* . These terms were queried in the PubMed and SciELO databases for articles published in English between January 2000 and July 2024. The figures are part of a personal collection of the authors, with publication consent obtained from all patients.

## NEURO-ONCOLOGY

Within the group of oncological manifestations, five disorders associated with an increased risk of neoplastic lesions will be discussed: NF1, NF2, TSC, VHL, and GGS. A variety of neoplastic lesions can occur in these syndromes, including low- and high-grade gliomas of the central nervous system, schwannomas, astrocytomas, hemangioblastomas, and multisystemic tumors. Skin evaluations may reveal diagnostic features, such as café-au-lait macules in neurofibromas NF1 and NF2, facial angiofibromas in TSC, and basal cell carcinomas in GGS. Early diagnosis is crucial for effective screening and management of these neoplastic conditions.

### Neurofibromatosis type I

Neurofibromatosis type 1, also known as Von Recklinghausen disease, is a multisystemic autosomal dominant disorder caused by variants in the *NF1* gene (OMIM 613113, *neurofibromin 1* ). 3 While family history is common, ∼ 50% of cases result from de novo variants. The global prevalence is estimated at 1:3,000. 3

This disorder demonstrates significant phenotypic variability, even within the same family. 3 4 Optic pathway gliomas, benign neoplastic lesions predominantly seen in children, may result in neuro-ophthalmological symptoms. 4 Additional neurological manifestations include cognitive impairment, behavioral changes, headaches (19%), motor deficits (6.2%), hydrocephalus (5%), and epilepsy (4.7%). 5 6 7 Particularly, headache is frequently associated with gliomas (50%), syringomyelia (20%), and Chiari malformations (20%). 5 Among adults, there is a 10- to 50-fold increased risk of developing high-grade gliomas. 6 Vascular complications, such as Moyamoya disease, arteriovenous fistula, dolichoectasia, and other cerebrovascular abnormalities, may result in ischemic or hemorrhagic strokes. 6

Neurofibromatosis type 1 can rarely present with symmetric polyneuropathy (up to 2.3% of patients), which should be distinguished from compressive manifestation of neurofibromas on nerve roots and peripheral nerves. 6

**Figure 1:**
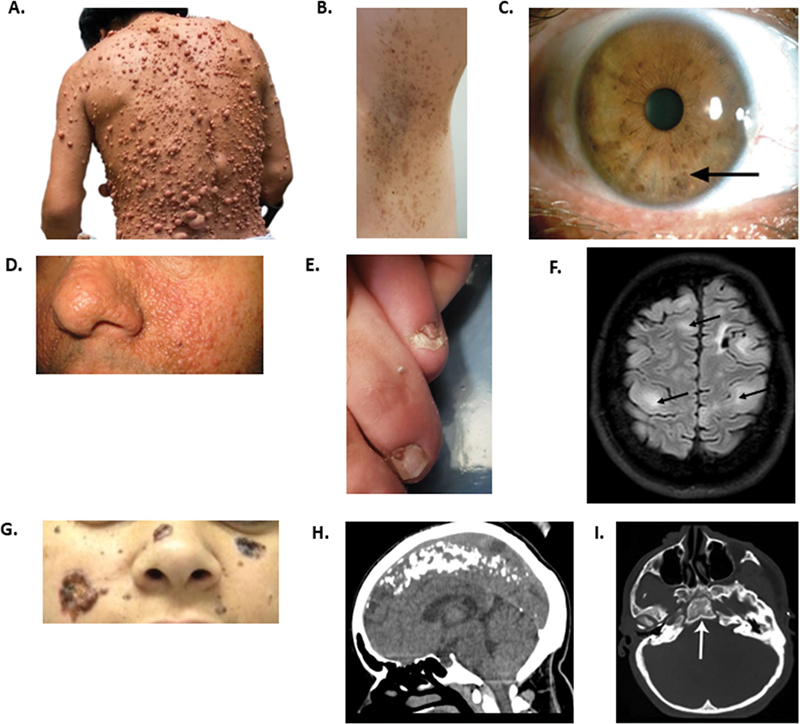
Clinical features of neoplastic group. ( **A–C** ) Neurofibromatosis type 1. A. Several neurofibromas on the trunk. ( **B** ) Freckling in the axillary. ( **C** ) Lisch nodules. ( **D–F** ) Tuberous sclerosis complex. ( **D** ) Facial angiofibromas. ( **E** ) Ungual fibromas. ( **F** ) Brain magnetic resonance imaging with multiple cortical tuberous. ( **G,H** ) Gorlin-Goltz syndrome. ( **G** ) Multiple nevi and basal cell carcinomas. ( **H** ) Brain computed tomography reveals cerebellar tent calcification. (I) Hypoplasia of the lower wing of the sphenoid bone.

Cutaneous manifestations, including café-au-lait macules and freckling in axillary and inguinal regions, are hallmark features. 7 Other key findings include subcutaneous and plexiform neurofibromas, malignant peripheral nerve sheath tumors, Lisch nodules, and skeletal dysplasia ( Figure 1A–C ). 4 5 6 7

Brain magnetic resonance imaging (MRI) often reveals hyperintensities on T2-weighted sequences, with either iso- or mildly increased signal on T1, typically without contrast enhancement. 6 These lesions are referred to as focal areas of signal intensity (FASI), which represent myelin vacuolization. 6 7 8 They are observed in 43 to 93% of children with NF1 and exhibit dynamic characteristics, with the potential to increase, decrease in size, or resolve over time. 9 However, they typically regress by adulthood (rarely in patients older than 20 years). 9 Therefore, an increase in the number or size of lesions in this age group should raise suspicion for neoplasia. 6 7 8 9 Furthermore, the presence of T1 hypointense lesions, contrast enhancement, or mass effect suggests glioma. 6 Magnetic resonance imaging follow-up and proton magnetic resonance spectroscopy may also be useful in distinguishing FASI from tumors. 8

Diagnosis is based on the Revised Diagnostic Criteria for Neurofibromatosis Type 1 and Legius Syndrome ( **Supplementary Material - Table S1** – available at https://www.arquivosdeneuropsiquiatria.org/wp-content/uploads/2025/04/ANP-2024.0378-Supplementary-Material.docx [online oly]). 10 An important differential diagnosis is Legius syndrome, an autosomal dominant condition caused by pathogenic variants in the *SPRED1* gene. 10 Unlike NF1, Legius syndrome lacks Lisch nodules and neurofibromas, although up to 50% of patients meet NF1 diagnostic criteria. 10

**Table 1:** Summary of neurocutaneous syndromes based on their neurological manifestations

	Disease	Gene	Inheritance	Neurology	Dermatology	Other clinical findings
Oncology	Neurofibromatosis type I	*NF1*	AD (50% de novo)	Optic pathway gliomas. Cerebrovascular disease	Neurofibromas. *Café-au-lait* macules. Axillary or inguinal freckling.	Lisch nodules. Long bone dysplasia. Sphenoid wing dysplasia.
	Neurofibromatosis type II	*NF2*	AD	Schwannomas. Meningiomas. Ependymomas. Astrocytomas. Neurofibromas. Peripheral neuropathy.	Schwannomas. Neurofibromas. *Café-au-lait* macules. (30%)	Juvenile cataract.
	Tuberous sclerosis	*TSC1* *TSC2*	AD	Cortical tuber. Subependymal nodule. SEGA. Infantile spasms and focal seizures. Cortical dysplasia. SEGA	Facial Angiofibromas. Hypomelanotic macules. Shagreen patches. Ungual fibromas.	Retinal hamartomas. Cardiac rhabdomyoma. Lymphangioleiomyomatosis. Angiomyolipomas.
	Von Hippel-Lindau	*VHL*	AD	Hemangioblastomas.	–	Retinal hemangioblastoma. Clear cell renal cell carcinoma. Pheochromocytomas; paragangliomas. Pancreatic neuroendocrine tumors.
	Gorlin-Goltz syndrome	*PTCH1*	AD (30% de novo)	Medulloblastoma. Macrocephaly. Calcified falx cerebri.	Basal cell carcinoma. Palmar or plantar pits. Epidermal cysts.	Odontogenic keratocysts. Calcified ovarian fibromas. Cardiac fibromas. Rib anomalies.
Epilepsy	Neurocutaneous melanosis	*NRAS*	Mosaicism	Melanosis or melanoma in the parenchyma or leptomeninges. Hydrocephalus.	Large or multiple congenital nevi.	–
	Hypomelanosis of Ito	–	Mosaicism	Cognitive impairment. Behavior abnormalities. Cortical malformations including hemimegalencephaly.	Hypopigmented lesions along the Blaschko lines. Alopecia.	Musculoskeletal disturbances. Congenital cardiac disease.
	Lipoid proteinosis	*ECM1*	AR	Horn-shaped symmetric calcifications in temporal lobes. Memory impairment. Behavior abnormalities. Paresthesias.	Moniliform blepharosis. Skin nodules. Acneiform scars. Hyperkeratotic lesions on extensor surface.	Hoarseness.
Vascular	Sturge–Weber syndrome	*GNAQ*	Mosaicism	Intracranial angiomatosis	Port wine stains	Optical atrophy. Glaucoma
	Hereditary hemorrhagic telangiectasia	*ENG* *ACVRL1* *SMAD4*	AD	Arteriovenous malformations (AVMs)	Telangiectasias are in the face, hands, and oral cavity.	Nose bleeds
Ataxia	Ataxia-telangiectasia	*ATM*	AR	Early-onset ataxia. Ocular abnormalities. Extrapyramidal symptoms. Neuropathy.	Telangiectasias (bulbar conjunctiva, ears and face).	Immunodeficiency. Malignancy (especially hematological).
	Cerebrotendinous xanthomatosis	*CYP* *27A1*	AR	Intellectual disability. Cerebellar ataxia. Peripheral neuropathy. Pyramidal and extrapyramidal signs.	Tendon xanthoma.	Early-onset diarrhea. Bilateral juvenile cataracts.

Abbreviations: AD, autosomal dominant; AR, autosomal recessive.

Management includes surgical excision or laser ablation of neurofibromas and supportive measures such as emollients and psychological counseling. 11 Malignant peripheral nerve sheath tumors require complete resection with clear margins. 11 Selumetinib, a mitogen-activated protein kinase (MEK) kinase inhibitor, is an emerging treatment option for inoperable plexiform neurofibromas. 12 Regular monitoring is essential for detecting neoplastic or systemic complications. 11

### Neurofibromatosis type II

Neurofibromatosis type 2 is an autosomal dominant neoplasia predisposition syndrome caused by variants in the *NF2* gene (OMIM 607379, neurofibromin 2), which encodes the tumor suppressor protein merlin. 13 Its prevalence is ∼ 1:100,000. 13

Symptoms typically manifest from childhood to the third decade of life and include hearing loss, tinnitus, vertigo, and imbalance, often resulting from vestibular schwannomas, which occur in up to 95% of patients. 13 14 Schwannomas may also affect cranial, spinal, and peripheral nerves. 14 Other common neoplasms include meningiomas and ependymomas; less commonly, astrocytomas and neurofibromas may occur. 14

Dermatological findings include skin tumors (59–68%), skin plaques (41–48%), subcutaneous tumors (43–48%), café-au-lait macules (33–48%)—which are fewer in number than those seen in NF1, and hyperpigmented plaques. 13 Ophthalmologic manifestations, such as juvenile cataracts and epiretinal membranes, are also frequent. 15 Epiretinal membranes and retinal hamartomas may also occur. 13

Diagnosis is based on the Updated Diagnostic Criteria for NF2 and Schwannomatosis (2022) ( **Supplementary Material Table S2** [online oly]). 16 Management typically involves surgical resection of vestibular schwannomas. 13 For tumors smaller than 3 cm, hearing preservation is achievable in 65% of patients, although surgical risks increase with tumor size. 13 Brigatinib, a derivative of anaplastic lymphoma kinase inhibitor-1 (ALK-IN-1), has emerged as a potential treatment for NF2-associated tumors. 17

**Table 2:** Differential diagnosis of neurocutaneous syndromes based on dermatological abnormalities and tumors

Dermatological abnormalities		
Hyperpigmentation	Hypopigmentation	Nodules
Cafè-au-lait spots______________NF1 Freckling_____________________NF1 Congenital melanocytic nevi______NCMS	Ash-leaf macules_________TSC Blaschko line____________HI	Skin nodules___________________NF1/NF2/LP Skin plaques___________________NF2/TSC Proliferative melanocytic nodules___NCMS
Tumors		
Neural tumors		Non-neural tumors
Optic pathway gliomas________________NF1 Gliomas-astrocytomas________________NF1/NF2 Vestibular schwannomas______________NF2 Non-vestibular schwannomas__________NF2/NF1 Meningiomas_______________________NF2/NF1 Hemangioblastomas_________________VHL Medulloblastomas___________________Gorlin Cortical tubers______________________TSC Subependymal nodules_______________TSC SEGA_____________________________TSC Brain tumors________________________NCMS		Facial angiofibromas____________________________TSC Gingival fibromas_______________________________TSC Basal cell carcinomas____________________________Gorlin Melanoma_____________________________________NCMS/Gorlin Cardiac rhabdomyomas__________________________TSC Pulmonary lymphangioleiomyomatosis_______________TSC Hamartomatous rectal polyps______________________TSC Retinal & non-renal hamartomas____________________TSC Renal angiomyolipomas___________________________TSC Renal cell carcinomas_____________________________TSC Pheochromocytomas______________________________VHL/NF1 Paragangliomas__________________________________VHL Pancreatic neuroendocrine tumors_____________________VHL Endolymphatic sac tumors____________________________VHL

Abbreviations: HI, hypomelanosis of Ito; LP, lipoid proteinosis; NCMS, neurocutaneous melanosis; NF1, neurofibromatosis type 1; NF2, neurofibromatosis type 2; TSC, tuberous sclerosis complex; VHL, Von-Hippel-Lindau.

### Tuberous sclerosis complex

Tuberous sclerosis complex is an autosomal dominant disorder caused by variants in the *TSC1* (OMIM 605284, hamartin) and *TSC2* (OMIM 191092, tuberin) genes. 18 These genetic alterations lead to the development of multisystemic hamartomas. 18 Although TSC follows an autosomal dominant inheritance pattern, approximately two thirds of cases arise sporadically. 19 Variants in the *TSC1* and *TSC2* genes result in dysfunctional forms of the proteins hamartin and tuberin, respectively. 19 This dysfunction impairs the suppression of tumor growth by disrupting signaling in the mammalian target of rapamycin (mTOR) pathway. 20 Tuberin variants, which account for 75 to 80% of sporadic cases, are particularly associated with more severe phenotypic expressions. 19 The prevalence of TSC is estimated to be 1 in 6,000 to 10,000 individuals, with an equal distribution across genders and ethnicities. 18

Tumors are a hallmark of TSC, including cortical tubers, subependymal nodules, subependymal giant-cell astrocytomas (SEGAs), facial angiofibromas, retinal and non-renal hamartomas, hamartomatous rectal polyps, cardiac rhabdomyomas (especially in fetuses and neonates), pulmonary lymphangioleiomyomatosis and gingival fibromas. 19 21 Patients may also develop renal angiomyolipomas, multiple renal cysts and renal cell carcinoma. 19

Neurologically, TSC is characterized by epilepsy, cognitive impairment, autism spectrum disorder and psychopathological issues. 19 Skin manifestations are found in nearly all individuals with TSC and include facial angiofibromas (75%), ash-leaf macules (90%), confetti lesions (small hypopigmented macules), shagreen patches (thickened skin areas on the lower back in over 50%), and ungual fibromas (20–80%) ( Figure 1D,E ). 21 Dental pits, seen in 90% of patients, are a hallmark feature of TSC, compared with just 9% in the general population. 19

The diagnosis of TSC is based on criteria established by the second International Tuberous Sclerosis Complex Consensus ( **Supplementary Material Table S3** [online oly]). 22 A definitive diagnosis requires meeting two major criteria or one major criterion in combination with two minor criteria. 22 The identification of pathogenic variants in the *TSC1* or *TSC2* genes is an independent diagnostic criterion. 18 Brain MRI often reveals cortical tubers, white matter abnormalities, cortical dysplasia, radial migration lines, cyst-like white matter lesions, and subependymal nodules ( Figure 1F ). 20 22 In the first 2 decades of life, 10 to 15% of individuals with TSC may develop SEGAs. 21 23

The prognosis for individuals with TSC varies depending on the severity of their symptoms. 21 Early and effective seizure control is crucial, as delays in treatment can adversely affect developmental outcomes. 20 Vigabatrin is recommended as a first-line treatment for partial seizures and infantile spasms in infants, while neurosurgical resection is preferred for managing SEGAs. 21

### Von Hippel-Lindau disease

Von Hippel-Lindau is an autosomal dominant disorder that predisposes individuals to the development of multisystemic neoplasms. 24 It is caused by pathogenic variants in the *VHL* gene (OMIM 608537), a tumor suppressor located on the short arm of chromosome 3, which encodes the VHL protein. 24 The incidence of VHL is ∼1 in 36,000 live births. 24

This condition predisposes individuals to tumors in the brain, spinal cord, kidneys, adrenal glands, pancreas, and reproductive organs. 25 The most common tumors include nervous system and retinal hemangioblastomas, clear cell renal cell carcinoma, pheochromocytomas, paragangliomas, pancreatic neuroendocrine tumors, and endolymphatic sac tumors. 25 Hemangioblastomas occur in up to 80% of patients with VHL, typically manifesting during the second decade of life. 24 These tumors are frequently located in the cerebellum (70%), spinal cord (53%), and brainstem (22%), often causing symptoms such as ataxia or headache due to mass effect and, in some cases, non-communicating hydrocephalus. 24

Magnetic resonance imaging (MRI) typically reveals enhancing solid lesions or mural nodules accompanied by adjacent non-enhancing cysts. 26 Diagnosis is based on a combination of clinical and genetic criteria. 26 27 Clinical criteria are fulfilled when a patient with a first-degree relative diagnosed with VHL presents with at least one characteristic neoplastic manifestation, such as hemangioblastoma of the retina or central nervous system, renal cell carcinoma, pheochromocytoma, pancreatic neuroendocrine tumor, or endolymphatic sac tumor. 27

Management of symptomatic hemangioblastomas generally involves surgical resection, often preceded by preoperative embolization for larger lesions. 24 In asymptomatic patients, a watchful waiting approach is recommended, as nearly 50% of these lesions remain stable over a 5-year period. 24

### Gorlin-Goltz syndrome

Gorlin-Goltz syndrome, also known as basal cell nevus syndrome or nevoid basal cell carcinoma syndrome, is an autosomal dominant cancer predisposition disorder caused by pathogenic variants in the *PTCH1* gene (OMIM 109400), which encodes the transmembrane receptor protein patched homolog 1. 28 29 The prevalence of GGS ranges from 1:57,000 to 1:256,000 individuals and de novo variants account for up to 30% of cases. 28

Gorlin-Goltz syndrome is characterized by multiple basal cell carcinomas, as well as skeletal, ophthalmic, and neurological abnormalities ( Figure 1G ). 29 30 Up to 80% of patients develop basal cell carcinomas, ranging from tens to hundreds of lesions, primarily on the face, back, and chest. 29 30 Medulloblastoma and ovarian fibromas may also occur. 29 30 Neurological manifestations include nystagmus, intellectual disability, dural calcifications, bridging of the sella, agenesis of the corpus callosum, and congenital hydrocephalus. 29 30 A notable imaging finding is calcification of the falx cerebri and cerebellar tent, as well as hypoplasia of the lower wing of the sphenoid bone ( Figure 1H,I ). 31

The diagnosis of GGS follows criteria outlined in the Updated 5th Edition of the World Health Organization Classification of Head and Neck Tumors, requiring the presence of either 2 major criteria or one major and 2 minor criteria ( **Supplementary Material Table S4** [online oly]). 32

Given the multisystemic nature of the syndrome, a multidisciplinary approach is essential for its management. 28 29 30 This should involve coordinated care among specialists in orthopedics, dermatology, oncology, neurology, and neurosurgery. 28 29 30

## EPILEPSY

In this group, three neurocutaneous syndromes characterized by epilepsy as the main neurological presentation will be discussed: neurocutaneous melanosis (NCMS), HI, and LP. These disorders typically present with a range of seizure types, including infantile spasms, focal seizures, and bilateral tonic–clonic seizures. 19 33 34 35 They may also be accompanied by other neurological features, such as behavioral abnormalities and developmental delays. 19 33 34 35

Dermatological findings provide important diagnostic clues for these conditions. Congenital melanocytic nevi are characteristic of NCMS, hypopigmented lesions following the Blaschko lines suggest HI, and nodular facial lesions are indicative of LP. Early diagnosis and treatment are essential to improve developmental outcomes and manage seizures effectively in individuals with these syndromes. Epilepsy can also be secondary to tumors observed in the neurocutaneous syndromes of the neuro-oncology group.

### Neurocutaneous melanosis

Neurocutaneous melanosis is characterized by multiple or large congenital melanocytic nevi (LCMN) on the skin, associated with the accumulation of melanocytes in the central nervous system (CNS). 33 The condition results from somatic mosaicism in the *NRAS* gene (OMIM 164790), leading to constitutive activation of *NRAS* and downstream signaling pathways. 36 The prevalence is unknown, and there is no sex predilection. 37 38

Neurological involvement typically includes melanosis in the brain parenchyma and leptomeninges, presenting with seizures, developmental delay, psychiatric symptoms, or sometimes being asymptomatic. 37 Epilepsy is the most common manifestation, including bilateral tonic-clonic, myoclonic, or focal seizures. 39 The prognosis for epilepsy is most strongly predicted by the localization of parenchymal melanosis, particularly if it involves the amygdala. 39 The most severe complication of NCMS is intracranial hypertension due to communicating hydrocephalus. 39 Brain tumors, either primary or metastatic (e.g., melanoma), are also common. 39 Less frequently, NCMS can present with myelopathy, ataxia, hemiparesis, or cranial neuropathies (especially cranial nerves VI and VII). 33 38 39

**Figure 2:**
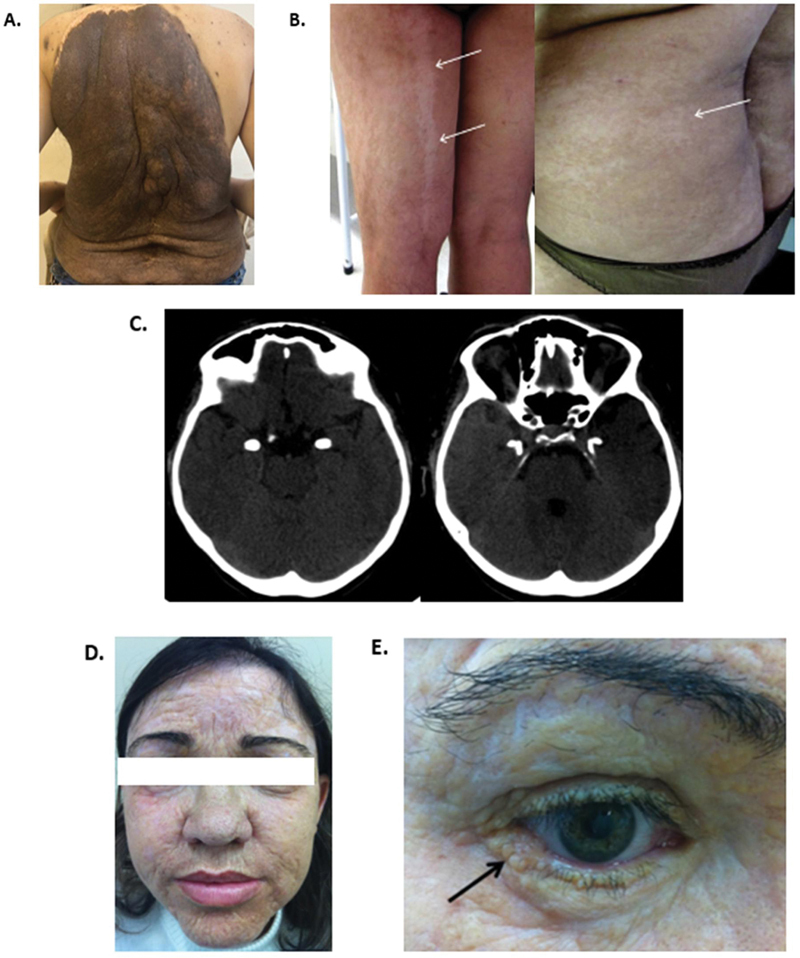
Dermatological and radiological manifestations of epilepsy group. ( **A** ) Neurocutaneous melanosis - large congenital melanocytic nevi affecting the trunk in a “bathing suit” pattern. ( **B** ) Hypomelanosis of Ito - hypopigmented lesions along the Blaschko lines. ( **C–E** ) Urbach-Wiethe disease. ( **C** ) Brain computed tomography with horn-shaped symmetric hippocampal calcification. ( **D** ) Diffuse thickening of the skin. ( **E** ) Moniliform blepharosis.

Dermatological manifestations include benign congenital melanocytic nevi (CMN), proliferative melanocytic nodules, and melanoma ( Figure 2A ). 38 Congenital melanocytic nevi often involve the trunk in a “bathing suit” pattern, with other typical patterns including “shoulder stole” and “life-vest jacket” distributions. 39 The risk of NCMS in patients with LCMN ranges from 1 to 12%. 38

Diagnostic criteria include large or multiple congenital nevi (≥ 20 cm in adults, ≥ 9 cm on the head in neonates or infants, ≥ 6 cm on the body) associated with meningeal melanosis or melanoma. 38 Diagnosis requires exclusion of cutaneous and meningeal melanoma, unless benign histological lesions are present in both the skin and meninges. 38

Magnetic resonance imaging findings may show two distinct patterns: parenchymal (hyperintensity on T1-weighted images in the temporal lobes, amygdala, pons, and cerebellum) 38 40 and leptomeningeal (hyperintensity in the leptomeninges with diffuse gadolinium enhancement). 40 Radiological signs suggestive of malignancy include mass effect, edema, hemorrhage, necrosis, or nodular/plaque like enhancement. 40 Magnetic resonance imaging is not recommended for asymptomatic individuals. 37

Treatment includes anticonvulsant medications for epilepsy, with anterior temporal lobectomy or hippocampectomy considered for refractory cases. 39 Shunt insertion is recommended for hydrocephalus. 39

### Hypomelanosis of Ito

Hypomelanosis of Ito, also known as incontinentia pigmenti achromians, results from various forms of mosaicism rather than representing a distinct condition. 41 It affects ∼ 1 in 1,000 to 10,000 individuals, with no gender predilection. 42

The most common neurological presentations include epilepsy (37–53%), which typically manifests in the first year of life, and cognitive impairment (60%). 42 43 Seizures may present as infantile spasms, focal seizures with impaired awareness, myoclonic seizures, or bilateral tonic–clonic seizures. 42 43 Hypomelanosis of Ito has heterogeneous clinical presentation, including adult-onset dementia associated with enlarged Virchow-Robin spaces. 44

The dermatological hallmark of HI consists of hypopigmented lesions along the Blaschko lines, which represent fetal epidermal cell migration patterns ( Figure 2B ). 34 45 These lesions typically present as stripes (often ending at the midline) or in a patchy pattern, affecting the trunk and limbs, and are frequently associated with anhidrosis. 34 45 They are present at birth in 64 to 80% of patients and may diminish after adolescence. 45 Musculoskeletal manifestations are also frequent and include short stature, scoliosis, hemihypertrophy or hemihypotrophy, pectus carinatum or excavatum, and finger anomalies. 34

Diagnostic criteria for HI require one sine qua non criterion (congenital or early-acquired nonhereditary cutaneous hypopigmentation in linear streaks or patches affecting ≥ 2 body segments) plus at least one major or two minor criteria. 42 Major criteria include nervous system or musculoskeletal abnormalities, while minor criteria encompass ≥ 2 additional body malformations or chromosomal anomalies. 42

Radiological findings in HI include cortical malformations such as hemimegalencephaly, pachygyria, polymicrogyria, porencephaly, lissencephaly, cortical dysplasia, as well as hamartomas at the gray-white matter junction and arteriovenous malformations. 43

Treatment of HI involves managing seizures, with some patients requiring multiple anticonvulsant medications or even surgical interventions for effective control. 43 45

### Lipoid proteinosis

Lipoid proteinosis, also known as Urbach-Wiethe disease (UWD), is an autosomal recessive disorder caused by mutations leading to loss-of-function or reduced expression of the *ECM1* gene (OMIM 602201, extracellular matrix protein 1). 35 46 This results in the intracellular deposition of noncollagenous proteins and glycoproteins in multiple systems. Fewer than 100 cases of LP have been documented worldwide, and it affects both sexes equally. 35

Neurological manifestations include epilepsy, neuropsychiatric disorders, and migraine. 35 46 47 The most common form of epilepsy in LP is focal seizures, often characterized by impaired awareness with minimal or absent motor symptoms. 48

Dermatological features are key to the diagnosis, with interconnected bead-like papules on the eyelids (moniliform blepharosis) being a prominent sign, although it is present in only about half of individuals. 47 The skin may also show diffuse thickening in specific areas, along with papules, nodules, and plaques on the face and lips. 48 In more advanced stages, hyperkeratotic lesions may develop on the extensor surfaces of the arms and legs. 46 48 Additionally, two thirds of patients present with hoarseness, which typically appears at birth or early infancy, due to the early infiltration of hyaline material in the larynx, and tends to worsen over time. 47

An almost pathognomonic radiological finding of LP is symmetric bilateral hippocampal calcification seen on brain computed tomography (CT), which appears horn-shaped and involves the amygdala nuclei within the uncus of the temporal lobes ( Figure 2C–E ). 47 49 However, the presence and severity of calcification do not correlate with the occurrence of seizures. 48

Diagnosis can be confirmed through a skin biopsy and sequencing of *ECM1* variants. 48 Treatment is symptomatic, focusing on managing each symptom as it develops. 48

## VASCULAR

This section covers two disorders associated with vascular malformations: SWS and HHT. Port-wine stains, a hallmark of SWS, should raise suspicion for the diagnosis. Early identification of these skin lesions in newborns is crucial for ensuring timely neurological and ophthalmological follow-up. Rendu-Osler-Weber syndrome is associated with both hemorrhagic and ischemic strokes. It is an important condition to consider in cases of embolic stroke of undetermined origin, which will be described in this section.

### Sturge-Weber syndrome

Sturge-Weber syndrome is a rare genetic neurocutaneous disorder caused by somatic mosaicism variants in the *GNAQ* gene (OMIM 600998), which encodes a G-protein involved in transmembrane signaling. 50 51 The incidence of SWS is estimated to be between 1 in 20,000 and 1 in 50,000 live births. 51 52

**Figure 3:**
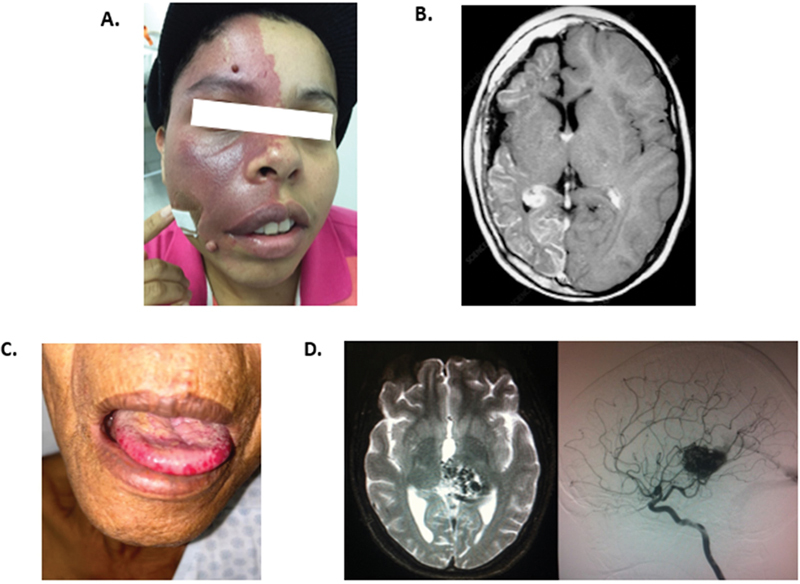
Clinical manifestations of vascular group. ( **A,B** ) Sturge Weber. ( **A** ) Port wine birthmark affecting the right trigeminal area. ( **B** ) Ipsilateral leptomeningeal vascular malformations with hemiatrophy. ( **C,D** ) Rendu-Osler-Weber. ( **C** ) Mucocutaneous telangiectasias. ( **D** ) Brain magnetic resonance imaging and digital subtraction angiography demonstrating arteriovenous malformation.

Neurological manifestations of SWS include capillary-venous leptomeningeal malformations and intracranial angiomatosis, typically affecting the occipital and parietal lobes. 53 Patients may present with seizures, hemiparesis, migraine-like headaches, delayed neuropsychological development, and stroke-like episodes. 54 Port-wine birthmarks (PWB) are a key feature, often present at birth, and can be unilateral, bilateral, or central ( Figure 3A,B ). 52 53 54 55 The most reliable indicator of SWS is the presence of PWB affecting the forehead, upper eyelid, and midline frontonasal prominence. 55 Over time, these marks may darken to a red or purple hue and persist throughout life. 55 Ocular manifestations include optic atrophy, glaucoma, and potential blindness. 54

Magnetic resonance imaging findings in SWS include leptomeningeal vascular malformations, abnormal venous drainage, reduced brain volume, enlarged ipsilateral choroid plexus, prominence of subependymal and medullary veins, and the characteristic tram-track sign due to cortical and subcortical calcifications. 50 51

Diagnosis of SWS requires at least two of the following: a facial port-wine birthmark, elevated intraocular pressure, and leptomeningeal angiomatosis. 51 According to the Roach scale, patients with isolated leptomeningeal angiomatosis (without skin or ocular involvement) can still be diagnosed as an intracranial form of SWS. 51 52 53 54 55

Treatment typically includes anticonvulsants such as carbamazepine or oxcarbazepine to control seizures, with surgical intervention for drug-resistant cases. 51 56 Glaucoma management may involve surgery for early-onset cases and topical medications. 51 Regular ophthalmological follow-up is essential for monitoring intraocular pressure and preventing optic nerve damage. 51

### Hereditary hemorrhagic telangiectasia

Hereditary hemorrhagic telangiectasia (HHT), also known as Rendu-Osler-Weber syndrome, is an autosomal dominant disorder characterized by multiple arteriovenous malformations (AVMs). 57 The condition is most commonly associated with variants in three genes: *ENG* (OMIM 131195, endoglin), *ACVRL1* (OMIM 601284, activin A receptor type II-like 1), and *SMAD4* (OMIM 600993). 57 58 The prevalence in North America is ∼ 1 in 10,000 individuals. 57

Neurological involvement includes brain AVMs, which are typically congenital and occur in ∼ 10% of patients, often leading to intracranial bleeding. 59 Spinal AVMs are rarer and may cause paralysis or back pain. 57 Lung AVMs can lead to right-to-left shunting, causing venous emboli to bypass the lungs and enter the arterial circulation, increasing the risk of transient ischemic attacks (TIAs), strokes, and brain abscesses, which are common complications in HHT patients. 59

Certain clinical features are age-related. 59 60 Epistaxis (nosebleed) affects 50% of patients by age 10 and up to 90% by age 21, with some individuals experiencing frequent episodes daily. 59 60 Telangiectasias are seen in 95% of adults, typically on the face, hands, and oral mucosa ( Figure 3C,D ). 59 These can also occur in the gastrointestinal tract, with up to 25% of patients presenting with digestive bleeding. 59 Hepatic vascular alterations are common (up to 74%), though they are rarely symptomatic. 59

Diagnosis is based on the Curacao 2000 criteria, which include the presence of epistaxis, mucocutaneous telangiectasias, visceral AVMs (pulmonary, cerebral, hepatic, gastrointestinal, and/or spinal), and a family history of the condition. 57 61 A definitive diagnosis is made when three or more criteria are met, possible with two criteria, and unlikely with just one. 57 61

Treatment is generally reserved for symptomatic telangiectasias affecting the skin, oral mucosa, gastrointestinal tract, and liver. 57 Brain and lung AVMs are usually treated when they present a high risk of complications. 57 Brain AVMs may be treated with embolization, neurosurgery, or stereotactic radiosurgery, while pulmonary AVMs are typically addressed with embolization. 57

## ATAXIA

This section describes two autosomal recessive disorders that present with early-onset ataxia: ataxia-telangiectasia (A-T) and cerebrotendinous xanthomatosis (CTX). Key diagnostic clues include systemic manifestations (e.g., early diarrhea and cataracts in CTX), non-ataxic neurological signs (such as pyramidal and extrapyramidal signs), and dermatological features (telangiectasias in A-T and tendinous xanthomas in CTX). Ataxia can also be secondary to tumors observed in the neurocutaneous syndromes of the neuro-oncology group.

### Ataxia-telangiectasia

Ataxia-telangiectasia (A-T) is an autosomal recessive disorder caused by mutations in the *ATM* gene (OMIM 607585, ataxia telangiectasia mutated) on chromosome 11q22. 62 The ATM protein is a tumor suppressor involved in DNA repair and genomic stability. Mutations in ATM impair its function, increasing the risk of cancers such as breast, prostate, and pancreatic cancer. Ataxia-telangiectasia syndrome is characterized by a predisposition to cancer and results from these mutations. 63 The prevalence of A-T is less than 1 in 100,000 live births. 64

**Figure 4:**
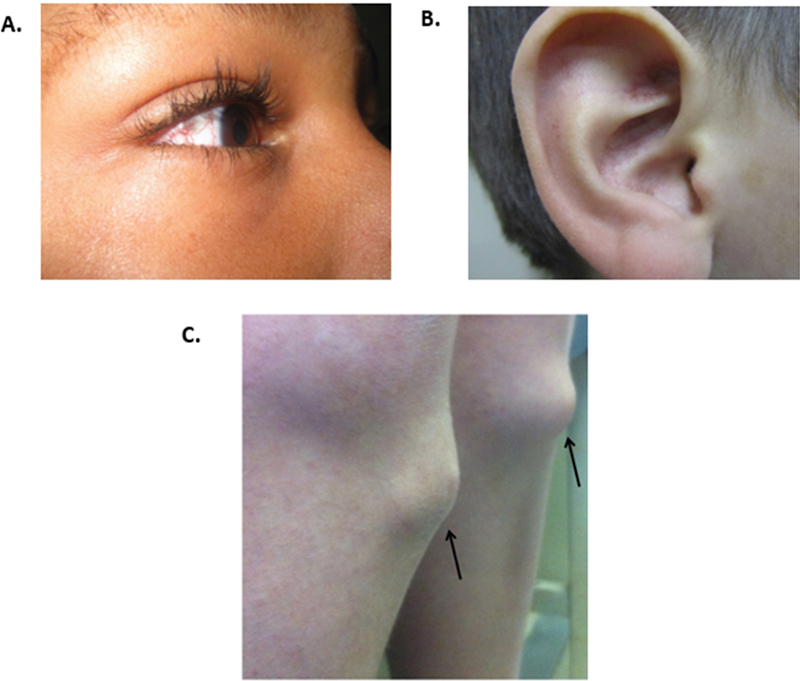
Dermatological features of ataxia group. ( **A,B** ) Ataxia-telangiectasia. Telangiectasias affecting the bulbar conjunctiva and the right ear. ( **C** ) Cerebrotendinous xanthomatosis – tendinous xanthomas located in the anterior tibial tuberosities.

Ataxia-telangiectasia typically presents as global ataxia in toddlers, marked by difficulties with sitting and gait, which remain relatively stable until around age. 62 As children age, symptoms progress, often requiring walking aids and leading to fine motor difficulties and extrapyramidal signs. 62 Additional neurological features include sensory-motor neuropathy and ocular abnormalities, such as oculomotor apraxia, nystagmus, strabismus, abnormal saccades, and vestibulo-ocular reflex deficits. After age 15, the condition tends to stabilize. 62 Asymptomatic telangiectasias, which typically appear before age 5, are commonly found in sun-exposed areas, particularly the bulbar conjunctiva (80–90%), ears, and face ( Figure 4A,B ). 63

Ataxia-telangiectasia is a systemic disorder with severe complications, including immunodeficiency, which leads to recurrent sinopulmonary infections, an increased predisposition to malignancies (especially lymphoid cancers), and radiation sensitivity. 62 63

Diagnosis of A-T is confirmed either by the absence of ATM protein or ATM kinase activity in cultured lymphocytes or skin biopsy samples, or by identifying pathogenic variants in the *ATM* gene through sequencing. 62 Magnetic resonance imaging often shows diffuse cerebellar atrophy, particularly involving the cerebellar vermis and hemispheres. 65 Additional supportive findings include elevated serum α-fetoprotein and hypogammaglobulinemia. 63

Prophylactic antibiotics are recommended for patients with a history of frequent recurrent infections (such as otitis media, sinusitis, bronchitis, and pneumonia), as well as for those with lymphopenia, to prevent opportunistic infections. 63 Ataxia-telangiectasia patients should be monitored for lung complications, such as bronchiectasis, and for exacerbations of symptoms. 63 Intravenous immunoglobulin (IVIg) therapy is also used to manage immunodeficiency. 63

### Cerebrotendinous xanthomatosis

Cerebrotendinous xanthomatosis (CTX) is an autosomal recessive disorder of lipid metabolism caused by pathogenic variants in the *CYP27A1* gene (OMIM 213700). 66 This results in reduced activity of sterol 27-hydroxylase, leading to the accumulation of lipids in tissues, particularly in the brain, eye lenses, and tendons. 67 68 The estimated prevalence of CTX is ∼ 1 in 50,000 individuals. 67

Neurological manifestations of CTX are heterogeneous and can include intellectual disability (often in the first decade of life), cerebellar ataxia (typically in the second to third decades; 36–83%), peripheral neuropathy (45%), movement disorders (such as parkinsonism, dystonia, myoclonus, and postural tremor; 87%), and pyramidal signs (64–92%). 68

The dermatological hallmark is tendon xanthomas, which typically appear in infancy and are commonly located on the Achilles tendons and tibial tuberosities, with possible involvement of the extensor tendons in the fingers and triceps ( Figure 4C ). 69 Nevertheless, tendon xanthomas are not pathognomonic and can also occur in other lipid metabolism disorders, such as familial hypercholesterolemia and sitosterolemia. 69 Early-onset diarrhea and bilateral juvenile cataracts are additional diagnostic clues that often appear before neurological symptoms. 68

If two of the following four clinical indicators are present (early cataracts, diarrhea, progressive neurological symptoms, and tendon xanthomas), biochemical testing should be performed to check for elevated serum cholestanol levels. 68 Magnetic resonance imaging typically shows T2-weighted hyperintensity in the dentate nuclei, which is the most distinctive feature of CTX, although abnormalities in the periventricular white matter, globus pallidus, and global brain atrophy may also be seen. 68 70 *CYP27A1* gene sequencing is recommended for all suspected cases. 68

Treatment of CTX typically involves chenodeoxycholic acid (CDCA), which is the standard therapy. 68 While CDCA does not significantly improve dermatological or ophthalmological manifestations, it can help stabilize or improve neurological symptoms. 68 Additionally, due to the risk of early atherosclerosis, regular cardiovascular follow-up is recommended for patients with CTX. 68

This review provides a practical classification of NCS based on their predominant neurological manifestations, including neoplastic lesions, epilepsy, vascular abnormalities, and ataxia. Nevertheless, some neurological manifestations, including seizures, cognitive impairment, and behavioral changes, are common across these disorders, emphasizing the importance of integrated multidisciplinary care.

We have summarized the key disorders discussed in this article, including the associated genes, inheritance patterns, as well as the neurological, dermatological, and systemic features ( Table 1 ). We also provided a graphic representation of the differential diagnosis of NCS based on dermatological abnormalities and tumors ( Table 2 ). Early recognition of key dermatological features, even in newborns—such as port-wine stains in SWS and LCMN in NCM—can prompt timely neurological evaluation. Skin manifestations in conditions like TSC, HI, and LP provide critical diagnostic clues that can improve patient outcomes when detected early. Systemic features also contribute to raising diagnostic suspicion, such as early-onset diarrhea and bilateral juvenile cataracts in cerebrotendinous xanthomatosis.

In summary, routine dermatological screening is essential for neurologists to facilitate early diagnosis and appropriate management of NCS, ultimately improving patient prognosis.
